# Diversity across organisational scale emerges through dispersal ability and speciation dynamics in tropical fish

**DOI:** 10.1186/s12915-023-01771-3

**Published:** 2023-12-05

**Authors:** Thomas Keggin, Conor Waldock, Alexander Skeels, Oskar Hagen, Camille Albouy, Stéphanie Manel, Loïc Pellissier

**Affiliations:** 1 Ecosystems and Landscape Evolution, Institute of Terrestrial Ecosystems, Department of Environmental Systems Science, ETH Zürich, Zurich, Switzerland; 2 Unit of Land Change Science, Swiss Federal Institute for Forest, Snow and Landscape Research WSL, Birmensdorf, Switzerland; 3 Division of Aquatic Ecology and Evolution, Institute of Ecology and Evolution, University of Bern, Bern, Switzerland; 4 Department of Fish Ecology and Evolution, Center for Ecology, Evolution and Biogeochemistry, Eawag - Swiss Federal Institute of Aquatic Science and Technology, Kastanienbaum, Switzerland; 5 Division of Ecology & Evolution, Research School of Biology, Australian National University Canberra, Canberra, Australia; 6 Evolution and Adaptation, German Centre for Integrative Biodiversity Research (iDiv) Halle-Jena-Leipzig, Leipzig, Germany; 7 Department of Ecological Modelling, UFZ–Helmholtz Centre for Environmental Research, Leipzig, Germany; 8 CEFE, Univ. Montpellier, CNRS, EPHE- PSL University, Montpellier, France; 9 Institut Universitaire de France, Paris, France

**Keywords:** Models/simulations, Species-genetic diversity correlations, Macroevolution, Ecology, Speciation, Dispersal

## Abstract

**Background:**

Biodiversity exists at different levels of organisation: e.g. genetic, individual, population, species, and community. These levels of organisation all exist within the same system, with diversity patterns emerging across organisational scales through several key processes. Despite this inherent interconnectivity, observational studies reveal that diversity patterns across levels are not consistent and the underlying mechanisms for variable continuity in diversity across levels remain elusive. To investigate these mechanisms, we apply a spatially explicit simulation model to simulate the global diversification of tropical reef fishes at both the population and species levels through emergent population-level processes.

**Results:**

We find significant relationships between the population and species levels of diversity which vary depending on both the measure of diversity and the spatial partitioning considered. In turn, these population-species relationships are driven by modelled biological trait parameters, especially the divergence threshold at which populations speciate.

**Conclusions:**

To explain variation in multi-level diversity patterns, we propose a simple, yet novel, population-to-species diversity partitioning mechanism through speciation which disrupts continuous diversity patterns across organisational levels. We expect that in real-world systems this mechanism is driven by the molecular dynamics that determine genetic incompatibility, and therefore reproductive isolation between individuals. We put forward a framework in which the mechanisms underlying patterns of diversity across organisational levels are universal, and through this show how variable patterns of diversity can emerge through organisational scale.

**Supplementary Information:**

The online version contains supplementary material available at 10.1186/s12915-023-01771-3.

## Background

Biological diversity is measured as variation within and between different levels of organisation; from nucleotides, genes, individuals, populations, and species, through to whole meta-communities [1]. The processes shaping diversity at these different organisational levels are often studied in isolation, despite inherently comprising a single, dynamic system. Efforts have been made to reconcile these disparate levels of biodiversity study [2–11], with processes such as gene-flow underpinning population divergence viewed as analogous to species dispersal underlying community divergence [6]. Inherent in this view is an assumption that fundamental and analogous processes operating at different organisational levels should generate analogous diversity patterns [2–15]. In contrast to this expectation, empirical studies show inconsistency in both the direction and the strength of diversity relationships between the genetic and species levels of organisation (positive [16–18], negative [19] or weak [20] relationships have all been documented). Interpreting and comparing these mixed results is complicated by methodological decisions such as differences in genetic markers (neutral vs selective, mitochondrial vs nuclear), and how genetic information, populations and communities are spatially aggregated for comparisons. With relatively few empirical studies to guide us forwards, we instead aim to roll back some of this complexity by presenting a conceptual and analytical framework built from first principals.

Previous studies are constrained to correlative approaches to assess the relationship between levels of organisation, i.e. correlations between genetic diversity and species diversity patterns [2–15], which can constrain our thinking. It is intuitive, but perhaps flawed, to measure patterns of diversity at each level of diversity (genetic and species), to infer how those patterns were formed through processes known independently at each level, and then to compare these processes. We might look at the genetic level and infer the contributions of genetic drift, selection, mutation, and gene-flow. Then we could do the same at the species-level — inferring the roles of dispersal, selection, and speciation. We could then compare the respective processes at each level. This assumes that because patterns are measured at distinct levels of organisation, the processes underlying them are equally separated. The resulting interpretation is often that patterns at each level of diversity are determined by parallel sets of analogous processes [6] which can feedback between organisational levels [7, 19]. However, if we consider that organisational levels comprise a single biological system (e.g. that species are aggregations of individuals and their respective alleles), it might become clear that these “parallel” processes appear analogous because they are, instead, the same thing. For example, consider the comparison between gene-flow at the population genetic level and dispersal between communities at the species-level. Population-level gene-flow is the reproductive result of the movement of individuals or their propagules between populations, whereas species-level dispersal is the movement and persistence of individuals or propagules between communities. Underlying both gene-flow and dispersal is the movement of individuals and their constituent alleles across a land- or seascape. There is a single process at play: individual dispersal. Similarly, drift is the stochastic change in the frequency of alleles within a population [12, 21] or species within a community [6]. Underlying both is a single process across both levels: random persistence of individuals and their constituent alleles. In population genetics, selection is the non-random survival of alleles in a population [15, 22]; in community ecology, selection is the non-random survival of species in a community [23]. Reduced down, there is only one process: the non-random fitness of individuals and their constituent alleles. An exception to this reduction of analogous processes is mutation and speciation: mutation and the subsequent generation of new genetic variants from existing biological material is a result of molecular replicative machinery and reproductive mode [14]. Similarly, speciation is the generation of diversity from existing material as a result of reproductive isolation between individuals [1]. We would posit that whilst similar in action and intrinsically linked, speciation occurs through sufficient genetic differentiation through mutation, drift, and selection to disrupt genomic compatibility [24], whilst mutation is the unreliable replication of genetic information over time [14]. Mutation within the constituent species of communities will have emergent effects on community dynamics [7]. Cessation of gene-flow as a result of speciation will have consequences for the individuals and alleles contained within both the parent and offspring species. Overall, we can deconstruct the analogous “parallel processes” of gene flow/dispersal, selection, drift, and mutation/speciation at each level of organisation into a more holistic framework (Table 1). Conceptually, the framework becomes simpler: there are unified processes that have consequences across multiple levels of organisation.

**Table 1:** Processes that have been described as analogous between levels of organisation, and their unified interpretation

Population genetic	Community ecology	Unified
Gene flow	Dispersal	*Individual dispersal*
Drift	Drift	*Random sampling of individuals*
Selection	Selection	*Non-random sampling of individuals*
Mutation		*Mutation*
	Speciation	*Speciation*

Mechanistic models provide an easily manipulated experimental environment to explore analytical and conceptual frameworks away from the complexity of observational study [25–28]. For example, population- and species-level patterns have been explored mechanistically at local patch scales, which found a neutral positive correlative expectation between organisational levels made variable by introducing selection. More recently, mechanistic models have included both deep-time evolutionary processes and shallow-time ecological processes alongside broad-scale environmental information, integrating eco-evolutionary dynamics more completely with landscape dynamics [26, 29–31]. This approach provides the opportunity to explore various processes including drift, dispersal, mutation, and speciation across a dynamic landscape within a unified modelling framework. In particular, the “gen3sis” engine explicitly simulates population-level processes across a dynamic land- or seascape, allowing both population and species-level diversity patterns to emerge through dispersal, population differentiation, trait mutation, and trait selection [32]. This mechanistic framework allows us to explore the emergence of population and species-level diversity patterns without assuming relationships between the two. The processes within the model are all executed at the population-level, i.e. there is only one set of processes generating emergent diversity patterns at both the population and species levels of organisation.

Island systems provide attractive model systems for investigating diversification as more discrete habitat patches provide a clearer definition of populations and communities [33]. Reef fishes are such a system, being mostly constrained to easily defined patches of shallow, warm water [34–36]. They are highly diverse and have a wealth of spatial, phylogenetic, and trait information available [37]. At the species-level, tropical reef-associated fishes have spatially structured diversity patterns, with a centre of diversity in the Indo-Australian Archipelago that roughly follows a longitudinal negative gradient away from this major hotspot [35, 38, 39]. Similarly, genetic diversity studies find that spatial diversity patterns relate to seascape structure, barriers to dispersal, historical effects, and dispersal abilities [40]. These population- and species-level diversity patterns have been investigated in this system showing mixed relationships. A spatial positive relationship was observed between per-species mitochondrial nucleotide diversity and total species richness in tropical Pacific fishes [17]. Similarly, a positive relationship between global mitochondrial nucleotide diversity and species richness across both freshwater and marine fishes was found — aggregating spatially and comparing combined nucleotide diversity across all species to total species richness [18]. A positive relationship between the population and species levels was also found in the Western Indian Ocean, but only in pairwise comparisons between sites (β-diversity) and not at the local or global scales (α- or γ-diversity) [41] — indicating that diversity patterns across organisational scale are likely further dependent on the spatial partitioning considered.

To explore a framework of emergent diversity patterns across an organisational scale through unified processes, and to generate expectations, we simulate the diversification of the Euteleost radiation over the last 200 million years using biological traits and palaeogeological information. We implement this in the spatially explicit eco-evolutionary simulation engine, gen3sis [32], and consider different measures of diversity (richness, phylogenetic diversity, and mean pairwise distance); and spatial partitioning (γ, the mean global diversity generated within the system, and β, the diversity dissimilarity between geographically distinct regions). From the emergent patterns, we aim to work through the following questions:

1. Across multiple facets of diversity, what are the emergent relationships between population and species levels of diversity?
2. Which population-level processes amongst dispersal, differentiation, mutation, selection, and speciation drive variation in population-species diversity relationships?
3. How do population-species diversity relationships relate to clade properties such as range size and endemism?

## Results

We varied model parameter values across model simulation runs, with each model simulation conceptually considered to be one clade of fish with the given parameters. These parameters define the clade’s biological traits and properties, and our simulations reproduced variation in diversity across these. From 15,000 simulations, 1540 were retained that contained 20 or more extant species (median = 55). There was a wide range of diversity values at both the species and population levels; in richness (species, 20–2893; population, 1–101), Faith’s phylogenetic diversity (species, 2752–1,182,687, population, 4–1693), and mean pairwise distance (species, 312–2308, population, 2–294). This variation was also true of diversity values across geographic regions and in clade properties such as species turnover and diversification rate both globally and regionally (Additional file 2: Table S1).

### Continuity across facets of diversity

**Fig. 1:**
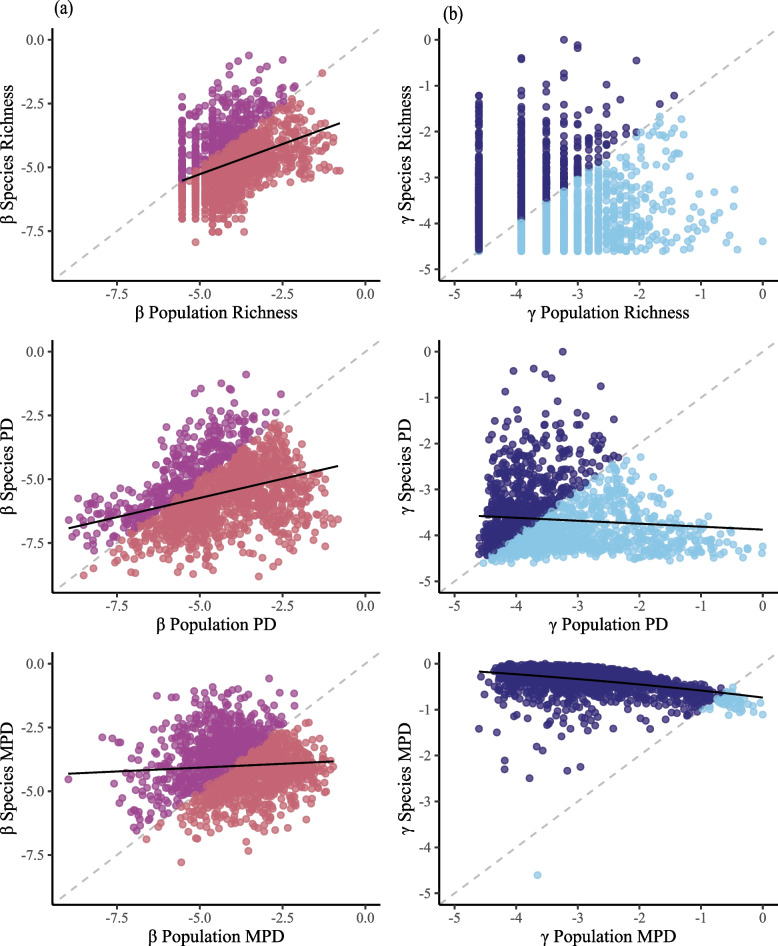
Simulated β- and γ-diversity relationships between the population and species levels of organisation across three measures of diversity; richness, phylogenetic diversity (PD), and mean pairwise distance (MPD). The grey dashed line represents a 1:1 positive relationship between the two levels, whilst the black solid lines represent the simulated relationship found through a significant (*p* < 0.05) simple linear regression. **a** All β-diversity relationships are positive, and **b** all but richness γ-diversity relationships are negative. Dark colours represent higher relative species diversity and lighter colours represent higher relative population diversity. All diversity measures have been log-transformed for both the regressions and visualisation. Figure data are available in Additional file 4

In all three diversity metrics, we found a negative relationship between γ-diversity at the population and species levels with effect sizes being greatest in mean pairwise distance (MPD), then phylogenetic diversity (PD), and finally richness which was not significant (richness, *β* <  − 0.01, *t* =  − 0.10, *p* = 0.92; PD, *β* =  − 0.06, *t* =  − 3.38, *p* < 0.05; MPD, *β* =  − 0.08, *t* =  − 20.14, *p* < 0.01; Fig. 1). In most retained simulations (96.6%), MPD values were relatively higher at the species-level than at the population-level, whilst richness and PD had a similar distribution of relative values at both the population and species levels (Additional file 1: Fig. S1). For measures of β-diversity, we found a positive relationship between the species and population levels (richness, *β* = 0.47, *t* = 16.34, *p* < 0.01; PD, *β* = 0.30, *t* = 15.01, *p* < 0.01; MPD, *β* = 0.06, *t* = 2.71, *p* < 0.01; Fig. 1). An increase in the difference between regions at the population-level was associated with an increase in the difference between regions at the species-level, with the strongest relationship occurring with the richness metric, then PD, followed by MPD.

### The impact of biological parameters on continuity

**Fig. 2:**
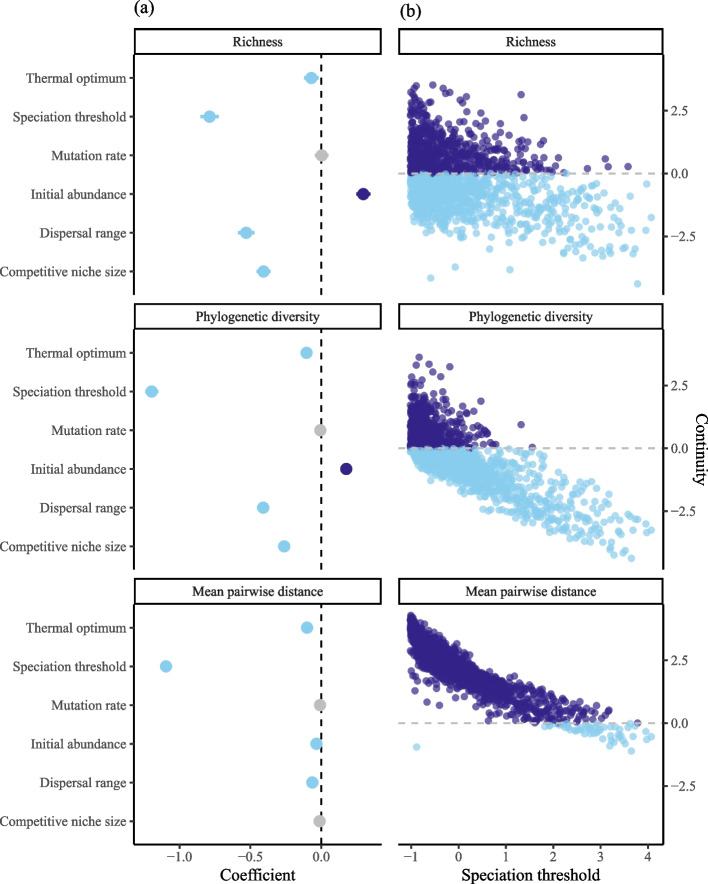
**a** Plots of multiple linear regression predictor coefficients showing the direction and magnitude of impact on population-species continuity metrics across each facet of diversity. Negative values (light blue) indicate that increasing the parameter value drives the relative diversity towards the population-level, whilst positive values (dark blue) drive diversity to the species-level. Horizontal bars represent the standard error. Greyed parameters are less significant (*p* > 0.05). **b** Scatterplot of continuity metrics against the most significant parameter, the speciation threshold. Positive values (dark blue) indicate relatively more species diversity and negative values (light blue) indicate relatively more population diversity. Figure data are available in Additional file 5

Continuity metrics of all three aspects of γ-diversity were significantly associated with biological parameters: richness (Adj. *R*^2^ = 0.35, *F* = 164.8, *p* < 0.001), PD (Adj. *R*^2^ = 0.66, *F* = 594.9, *p* < 0.001), and MPD (Adj. *R*^2^ = 0.83, *F* = 1834, *p* < 0.001). Higher values of our continuity metric indicate higher species diversity relative to population diversity, for example, in the case of richness higher continuity means fewer populations per species. For each parameter, a positive coefficient indicates that increasing a parameter increases the amount of species diversity relative to population diversity. Conversely, a negative coefficient indicates that increasing a parameter value increases the amount of population diversity relative to species diversity. The speciation threshold parameter had a consistently strong negative relationship across all three diversity continuity metrics (richness, *β* =  − 0.79, *t* =  − 23.99, *p* < 0.001; PD, *β* =  − 1.20, *t* =  − 51.02, *p* < 0.001; MPD, *β* =  − 1.09, *t* =  − 70.52, *p* < 0.001; Fig. 2). The parameters dispersal range, speciation threshold, and competitive niche size had a negative relationship with richness continuity (Fig. 2; Additional file 2: Table S2), whilst initial colonisation abundance had a positive relationship (*β* = 0.30, *t* = 11.84, *p* < 0.001). The parameters dispersal range, speciation threshold, competitive niche size, and thermal optimum had a negative relationship with PD continuity (Fig. 2; Additional file 2: Table S2), whilst the initial colonisation abundance had a positive relationship (*β* = 0.18, *t* = 9.87, *p* < 0.001). Speciation threshold, dispersal range, initial colonisation, and starting thermal optimum (Fig. 2; Additional file 2: Table S2) were negatively related to MPD continuity. The trait mutation rate was found to not be significantly associated with each of the three measures of continuity in diversity and was removed from all the models in the stepwise variable selection.

### Association of continuity with clade properties

**Fig. 3:**
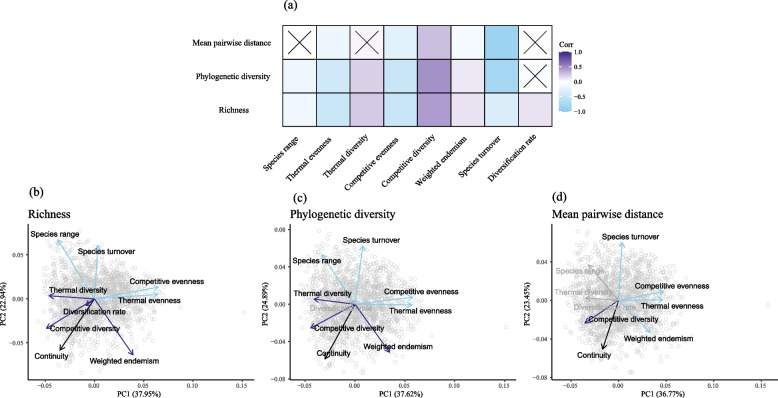
**a** Plot of the correlations between diversity continuity metrics and clade properties. Light blue indicates that increasing the clade property value is associated with an increased relative population-level diversity compared to species-level diversity, and vice versa for dark blue. Crosses indicate non-significant (*p* > 0.05) values. **b-d** PCA plots of each continuity metric and clade properties. Dark blue arrows indicate a significant (*p* < 0.05) correlation between the clade property and a relative increase in species-level to population-level diversity. Light blue indicates a significant correlation in the opposite direction. Grey clade properties had no significant relationship with the continuity metric. Figure data are available in Additional file 6

The relationships between the clade properties and each continuity metric were evaluated with pairwise Spearman’s rank correlations and visualised with a principal components analysis (PCA) for each facet of diversity (Fig. 3). For richness, increasing thermal evenness (*r *=  − 0.47, *p* < 0.01) and competitive evenness (*r* =  − 0.49, *p* < 0.01), and species turnover (*r* =  − 0.33, *p* < 0.01) were correlated with increasing population diversity relative to species diversity. The converse was true for thermal diversity (*r*(*n* = 1540) = 0.22, *p* < 0.01), competitive diversity (*r* = 0.42, *p* < 0.001), weighted endemism (*r* = 0.12, *p* < 0.01), and diversification rate (*r* = 0.12, *p* < 0.01), which were associated with an increase in species diversity relative to population diversity. These patterns were similar for the phylogenetic diversity and mean pairwise distance aspects of diversity (Additional file 2: Table S3). The differences were a lack of significant relationship between diversification rate and phylogenetic diversity (*r* =  − 0.05, *p* = 1.75), and no significant relationship between mean pairwise distance and all three of diversification rate (*r* = 0.01, *p* = 34.04), species range (*r* = 0.00, *p* = 47.41), and thermal diversity (*r* = 0.04, *p* = 4.83; Additional file 2: Table S3). There were no significant relationships between continuity across levels and species range, weighted endemism, and diversification rate (Additional file 2: Table S3).

In the PCA for all three diversity metrics, the first component accounted for between 36 and 38% of the variance, whilst the second component accounted for between 22 and 25% of the variance. For richness, the first component was contributed to mostly by both competitive and thermal evenness, followed by competitive and thermal diversity (Additional file 2: Table S4). Whilst the second component was mostly contributed to by species range, species turnover, and weighted endemism (Additional file 2: Table S4). For phylogenetic diversity, thermal and competitive diversity and evenness contributed most to the first component (Additional file 2, Table S4). Whilst the second component was contributed to most by species range, weighted endemism, and species turnover. Finally, for mean pairwise distance, the first component for mean pairwise distance was mostly contributed to by competitive and thermal evenness (Additional file 2: Table S4).

## Discussion

We used a spatially explicit simulation model to generate emergent patterns of population and species-level diversity through universal processes. We find that the strength and direction of the relationship between diversity at the population and species levels of biological organisation is variable and dependent on the diversity metrics considered. These results help lay a conceptual foundation to better understand widely different, and sometimes contradictory, patterns found in empirical data which are based on various metrics, spatial scales, and statistical aggregations [18, 19]. Specifically, we found a negative relationship between population and species diversity in γ-diversity metrics (total diversity at the population and species levels). This was most heavily determined by the speciation threshold — the amount of genetic divergence required to trigger speciation — determining the frequency of diversity partitioning from the population-level to the species-level. Conversely, we found that the population-species diversity relationship was positively correlated for β-diversity metrics, suggesting that geographic partitioning should emerge consistently through organisational scale. Finally, we describe the association between organisational continuity and clade traits which connects trait-based functional diversity measures [42, 43] to the emergence of contrasting diversity patterns across scales [18, 19, 44–46].

**Fig. 4:**
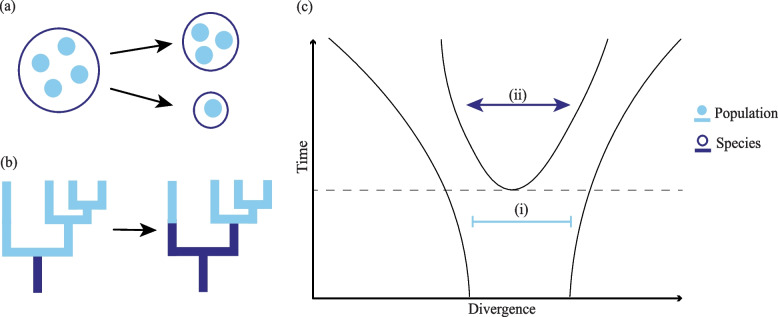
Conceptual diagram of the partitioning of diversity through speciation and the accumulation of divergence across levels of organisation. The species and population levels of organisation are represented by dark and light blue, respectively. **a** For richness, the total number of populations stays the same when speciation occurs, but another species is added to the system — 1 species and 4 populations becomes 2 species and 4 populations. This creates an uneven increase in the number of objects at each level. **b** Phylogenetic trees constructed from population objects are nested within species phylogenies. For phylogenetic diversity and mean pairwise distance, the phylogenetic tree topography, and therefore total diversity, is conserved throughout the speciation process. However, some of the population-level diversity is partitioned from the population-level to the species-level. Since speciation does not add or remove total diversity from the system, but rather transfers it directly from one to another, this dynamic drives a negative relationship between the species and population levels of diversity. **c** For divergence, (i) at the population-level the upper limit is determined by the speciation threshold and divergence is slowed by gene-flow and (ii) at the species-level there is no upper limit and few brakes inhibiting divergence between species over time. The dotted horizontal line represents a speciation event

Through simulating patterns of both population and species diversity through a set of universal processes, we show how population and species diversity are not necessarily positively related [47] and can even show negative relationships [19]. These patterns are difficult to explain through a framework that assumes levels of organisation should be driven by parallel processes [3, 5]. When considering the total global diversity (i.e. γ-diversity), we found negative relationships across two diversity measures: phylogenetic diversity (PD) and mean pairwise distance (MPD). This negative relationship was most strongly explained by the speciation threshold parameter. This dynamic was significant even when there was no significant correlation between levels of diversity (Figs. 1b and 2a). We infer that this negative relationship between species- and population-level diversity is mainly a consequence of a partitioning effect of the total diversity across the two levels of organisation (Fig. 4). In the simulation model, population-level diversity arises as populations migrate to new areas and eventually become isolated through environmental change. Eventually, isolated populations become new species at a rate modulated by the speciation threshold. Speciation does not remove diversity from the system, rather the diversity which was formerly between populations becomes diversity between species. As such, diversity has been directly transferred from the population-level to the species-level, decreasing the diversity at one level whilst increasing it at the other. This is supported by the strong negative relationship (the higher the speciation threshold, the more population diversity there is relative to species diversity) we find in our simulations between the speciation threshold and continuity in all three diversity metrics (Fig. 2b). Here, we infer that the time required for speciation to occur controls the rate at which diversity is partitioned between levels, with a shorter speciation threshold leading to a faster rate of partitioning. This model parameter is a proxy for several real-world interacting genomic processes underlying the accumulation of reproductive incompatibilities and eventual allopatric speciation of populations, such as mutation [48–50]. The rate in absolute time at which these reproductive incompatibilities accrue is determined by various traits such as generation time, background mutation rate, genomic architecture [51], and the complexity of life history traits [52–55] which are all inherited biological characteristics that vary across lineages [49, 56]. This suggests that the most important process in determining the emergence of diversity through the population and species levels of the organisation is even further down the scale of biological organisation: at the genome level.

The continuity between the population and species levels of diversity depended on the measure of biodiversity used (i.e. richness, PD, or MPD). As such, ignoring the multifaceted nature of diversity may overlook how common evolutionary mechanisms drive variation amongst biological levels of organisation [57]. As a metric, MPD is skewed heavily towards the species-level, with simulated clades typically having more divergence at the species-level relative to divergence at the population-level (Additional file 1: Fig. S1). The cause of this species-level skew in MPD, rather than PD, is likely driven by fundamental differences between populations and species in the aspect of diversity each metric is measuring. Phylogenetic diversity is a sum of the total branch length in a phylogeny and is heavily influenced by the number of objects present in the system (i.e. richness in populations or species; [57]), whilst the mean pairwise distance controls for this effect by averaging the number of objects and representing only the distances between them. The difference between PD and MPD is likely driven by two dynamics: the partitioning of diversity between organisational levels through speciation; and the homogenisation of populations through gene flow. Specifically, regarding PD, dispersal between populations shares alleles [12] and dispersal between communities shares species [58], homogenising the number of units present (richness) at both organisational levels. For MPD, on the other hand, some processes that decrease diversity at the population-level do not have a similar effect at the species-level. Dispersal between populations homogenises them through gene-flow, slowing divergence and therefore decreasing MPD values. At the species-level, dispersal between communities does not decrease species-species divergence (except for instances of introgression and horizontal gene transfer which are not explored in our model [59, 60]; Fig. 4c). Additionally reflected in MPD is that highly divergent populations eventually become new species — removing them from the population-level as they are partitioned into species-level objects through speciation. There is a population-level MPD limit, but not for species-level MPD (although this may be reduced considering evidence that evolutionarily distinct clades may be at higher risk of extinction [61], which may selectively remove highly divergent branches from the species-level phylogeny). This lack of removal of high divergence values between species allows species-level MPD to increase uninhibited. The result is two-fold: at the population-level, divergence is both capped by the speciation threshold and slowed by gene-flow; whilst at the species-level, divergence has few brakes and is limited only by increasing extinction probability over time, such that divergence values are limited to the sets,

1 $$\begin{array}{c}\nabla \text{species}=\left\{\uprho \le \nabla \text{species}<\infty \right\} \end{array}$$

2 $$\begin{array}{c} \nabla \text{population}=\left\{0\le \nabla \text{population}<\uprho \right\}\end{array}$$

where ∇ denotes divergence and ρ the speciation threshold (Fig. 4). These differing processes across measures of diversity highlight an important consideration in the study of continuity of diversity across organisational scale — we must be careful when comparing different organisational levels to ensure what we are measuring is actually comparable and be mindful that different metrics behave and interact in interestingly different ways across organisational scale.

Considering β-diversity (geographic dissimilarity) can highlight distinct patterns at both the population [62] and species levels [63] that differ to patterns in total (γ) diversity. In our simulations, the β-diversity metrics do not follow the same pattern as the γ-diversity metrics, with β-diversity values at the population and species levels showing a positive relationship (Fig. 1). The simulated positive relationships reflect those found in a tropical reef fish system which showed corresponding patterns between genetic differentiation and species turnover between sites in the Western Indian Ocean [41]. The cause is likely due to these β-diversity metrics being a measure of segregation of diversity across sites and is therefore scaled for the absolute variation in the system. This scaling makes β-diversity metrics a relative measure, and the partitioning effect of speciation on absolute diversity values should no longer apply. This allows patterns to form across levels, through processes such as drift, unimpeded. Our simulation results highlight the sensitivity of diversity measurement in understanding seemingly contradictory relationships between the population and species levels of organisation in empirical studies of these dynamics.

Biological traits modulate the eco-evolutionary processes that should in turn influence diversification across organisational scale [19, 64–66]. Dispersal range impacted continuity across all three facets of diversity (richness, phylogenetic diversity, and mean pairwise distance) with higher values driving more diversity at the population-level relative to the species-level. This is expected in an allopatric speciation model as higher dispersal increases range connectivity in a finite geographic space providing fewer opportunities for inter-population divergence to occur [32, 67]. Further, we explored how diversification across scales related to emergent clade properties. For example, high temporal species turnover is correlated with a larger population-level to species-level diversity ratio. This pattern relates to the idea that, unlike population diversity, species diversity is theoretically uncapped (Eqs. 1 and 2) — apart from the age of the simulation (or perhaps even real systems), there could be no hard limits to the maximum divergence between species, nor the complexity of their relationships [68]. In finite real-world systems, however, this may not be the case as limiters to species richness are well documented [26, 37, 69–71]. Our interpretation of the patterns found here is that extinction dynamics likely impact populations and species differently. The difference is the absolute values of diversity at each scale. Relatively, diversity takes longer to accrue to a maximum value at the species-level than the population-level (Eqs. 1 and 2). It follows that the relative diversity at the population-level can be generated back to its finite maximum (speciation threshold) much faster than to species can make it to a theoretically infinite maximum. This makes population diversity much more robust to extinction than it is at the species-level. These complex dynamics will be difficult to validate empirically, but we hope conceptualising them here is a first step in understanding how they develop across organisational scales.

## Limitations

We investigate the mechanisms driving diversification across organisational scale through a modelling approach for which there are clear limitations. The greatest is the spatial scale to which we are limited to γ- and β-diversity comparisons across organisations whilst in the knowledge that continuity in α-, β-, and γ-diversity behave differently [41]. In turn, we should also acknowledge that whilst our model is rooted in the real-world system of tropical reef-associated fishes, the goal is to meaningfully implement processes, not recreate patterns perfectly. Despite this, the mechanistic modelling approach applied here shows that even with a relatively simple representation of biological processes, observed patterns can broadly be reproduced (Additional file 1: Fig. S2) [72]. These include the Indo-Australasian Archipelago major hotspot, and Indian Ocean and Caribbean minor hotspots, as well as the latitudinal gradient of low equatorial richness followed by tropical increase and eventual temperate decrease [38, 72]. Key differences between simulated and observed patterns are likely a result of the model resolution and exclusion of key oceanographic dynamics. The low-resolution results in the Red Sea being isolated from the Indian Ocean and the Indo-Malayan archipelago fusing into an impermeable barrier. We decided to leave these inaccuracies that emerge in the final time step of our model in to remain more consistent with the accuracy of timesteps into the past. The simulation also did not account for the Eastern Pacific Barrier, the Benguela Current in the Eastern Atlantic which inhibits shallow water coral reef formation and dispersal, and the obstructive fresh-water outflow from major river basins [73]. Furthermore, it is likely the latitudinal gradient remained under-developed due to the hard temperature and depth limits used to compile the landscape inputs. This prevents potential back-and-forth colonisation of tropical reef fish clades to colder and/or deeper waters [74]. Given these considerations, we have confidence that the parameters and landscapes we did implement performed well in emulating process, and that these are viable for inferring the fundamental processes that shape diversity across organisational scale that we aimed to explore.

## Conclusions

We model the emergence of diversity from the population to species levels of biological organisation through a framework of universal eco-evolutionary processes. We posit the speciation threshold to be an important driver of the formation of counter-intuitive continuity in diversity patterns across organisational levels. In turn, this speciation threshold parameter is a proxy for a vast world of mechanisms below the population and species levels of organisation — at the scale of the individual and gene — indicating that to fully understand these patterns we must consider mechanisms across the full breadth of organisational scale and that our focus on population-to-species continuity in diversity patterns is only a start. We also highlight that patterns of continuity in diversity patterns across organisational scales are sensitive to the aspect of diversity measured and the metrics used. Finally, we uncover covariation between continuity in diversity across organisational scale and common ecological descriptors which we hope helps provide context for these dynamics in the larger field of eco-evolutionary study. In all, we hope the simulation methods here provide a useful conceptual and analytical framework, with associated expectations of emergent diversity patterns, for the holistic study of diversity formation through organisational scale.

## Methods

To model the diversification of tropical reef fishes, we used the mechanistic simulation engine, gen3sis [32]. Gen3sis is configured with species objects with information down to the population-level and runs over a spatially explicit landscape — which can be customised with biological configurations and paleoenvironmental reconstructions, respectively.

### Paleo-environmental reconstructions

As input, gen3sis requires both a physical landscape with which modelled species interact, and a distance matrix to determine the cost of dispersal across the landscape [32]. The landscape consists of marine bathymetry and sea surface temperature at a 1 × 1° resolution at 166.7 ka time steps back to 200 Ma [75, 76]. The extent of the input data is global, but habitable cells are restricted to those above a mean temperature of 17°C and shallower than 2000m. These cut-offs were chosen based on modelled thermal ranges of extant coral reef fishes [77] and visually matched with current coral reef distributions [78]. The distance matrices allow free movement in all marine cells and no movement across terrestrial cells.

Bathymetry was derived from an elevation model based on a mixture of plate tectonic modelling and geological evidence, described in detail by Scotese [75]. To match the model time steps here, these existing time steps were temporally interpolated using a linear function. Cells above sea level were removed. Temperature data are derived from a model based on oxygen isotope information, lithologic indicators, and the bio-geological record described in Scotese, Song [76]. As published, these data describe average tropical temperature change from the present (delta temperature) in 5 Ma time intervals into the past. These values are then modified geographically based on reconstructed climatic bands (paleo-Köppen belts). To generate one degree resolution sea surface temperature estimates, the boundaries of the climatic belts were first smoothed using the focal() function in the R raster package using a focal window of 81 cells. Boundary values for the north and south poles where the focal window exceeded the limits of the global extent were set to − 20 °C, matching the temperature values of the polar climate bands. From these smoothed 5 Ma intervals, smoothed spatial climate distributions were generated for each 166.7 ka time step using linear interpolation. Further, delta temperature values were calculated for each time step by linearly interpolating the 1 Ma interval values provided by and applied to the new geographically smoothed time steps. Finally, corrections were made to account for climatic fluctuations associated with recent glacial maxima [79]. Cost distance values between habitable cells in the reconstructed landscapes were calculated using the transition() function in the gdistance package in R [80]. The shortest path between each pair of cells was calculated and the distance between all pairs was stored in a distance matrix. Paths were calculated using an 8-direction adjacency scheme whereby cells are deemed adjacent if they are in contact vertically, horizontally, or diagonally. Each cell is also given a conductance value representing ease of travel across that cell. All marine cells were given a value of 1 (passable), whilst terrestrial cells were given a value of 0 (impassable).

### Biological configuration

For each species within our simulations, we store the values for species’ traits, abundance, and cell-to-cell differentiation across all inhabited cells in the species object. The species traits include a thermal optimum, a competitive niche value, and a niche width determining the competitive range of a population; these are summarised in Table 2. Each simulation was seeded with a single species occupying all habitable cells in the first time-step with the trait values described above and run with the following functions at each time step. The speciation threshold parameter represents allopatric speciation and is simulated through the use of divergence between geographically distinct adjacent cell clusters within a species. Geographic cells that experience no dispersal between them in a time step will increase their pairwise divergence by 1. Cells that experience dispersal will decrease their divergence by 1. If all the divergence values between two cells exceed the speciation threshold, then a new species will form. Conceptually, this is an abstracted model for genetic drift between spatially isolated populations, and homogenisation through gene-flow with successful dispersal events between them. The speciation threshold is then representative of allopatric speciation through genetic differentiation through isolation and drift.

**Table 2:** Summary of simulation parameters

*Parameter*	*Description*	*Parameter space*
*Initial abundance*	When a new cell is colonised, it is seeded with an initial abundance (whereafter the abundance returns to 1 with each time step).	**0.11–1**. From the minimum value before extinction to full abundance on colonisation.
*Thermal optimum*	The thermal optimum of the root species at the start of the simulation was varied across the entire temperature range present in all habitable cells across the entire simulation.	**17–31.4 °C**. Values from [77].
*Dispersal distance*	The distance a species can disperse from cell-to-cell at each time step. This determines inter-population connectivity and colonisation events. These values are taken from a Weibull distribution approximating the probability distribution of dispersal events.	The scale of the Weibull dispersal kernel was varied from **100 to 5000 km** based on long-term movement observations reported by [81] for non-pelagic coral reef fishes. The shape was set to 2.5.
*Speciation threshold*	The divergence threshold at which two populations will speciate.	**12–600 timesteps**, equivalent to between **20 ka and 1 ma**. The divergence required for two populations to allopatrically speciate is complex [49]. Here, we simply explore as wide a range of values as possible.
*Mutation rate*	The standard deviation of the normal distribution around the thermal and competitive nice traits from which new trait values are picked at each time step.	**0.01 to 0.15**. These values were based on estimation based on preliminary simulations.
*Competitive niche width*	The amount of competitive space around the competitive niche trait value within which other species will compete.	**0.02 to 0.50**. The competitive niche width was varied from 0.02 to 0.50 based on preliminary simulations.

Each time step, for every pair of inhabited cells, a potential dispersal event is calculated. The dispersal distance parameter is drawn from a Weibull distribution; if the dispersal distance exceeds the geographical distance between cells, the dispersal attempt is successful. On a successful dispersal attempt, if the target cell is already occupied, then the pairwise divergence value between those two cells is reduced, simulating gene-flow. If the target cell is unoccupied by that species, a colonisation event occurs. In the case of colonisation, the starting abundance is reduced to the initial abundance parameter value, allowing for incumbency effects.

Every time step, the competitive niche and thermal optimum of each species are subject to change. Firstly, the traits are modified by the addition of a random value drawn from a Gaussian distribution of mean 0 and a standard deviation that varies between simulations, but is common between traits. Once the traits of each species in each cell have been modified, traits of geographically adjacent clusters of cells within species are homogenised by assigning the mean trait values. The ecology function determines the abundance values [1] of each species within each cell. This is done through a simulation of temperature tolerance and competition. At the start of each time step, the abundance value is at the maximum of 1. It is then reduced based on the distance between the environmental temperature and the thermal optimum of the population. The reduction is proportional to the magnitude of the probability density of a Gaussian distribution function with a mean equal to the environmental temperature value and a standard deviation of 2 °C. Once the abundances of the species within a cell have been adjusted by abiotic factors, biotic interactions are carried out. Each species has a competitive niche value between 0 and 1, representing an abstract competitive space. They also have a competitive width value which determines the amount of that competitive space on either side of the niche value in which that species competes, e.g. if one species has a niche value of 0.3 and another with 0.4, and the competitive width is 0.2, then those two species will experience competition with one another. Species with overlapping niches will compete proportional to both their respective abundances and the size of the overlap. That is, a species with a high abundance will exert a greater competitive pressure than a species with a low abundance. Abundances are then also further reduced by the proportion of their competitive space that exceeds the 0–1 bounds. Finally, species whose abundances have been reduced to a value less than 0.1 are reduced to 0, causing local extinction in that cell.

Through modifying these parameters, we explored the impact of biological traits on the relationship between the species and population levels of organisation. This was done through varying the parameters summarised in Table 2 using tropical reef-fish values taken from the literature. Given the heavy nature of the model, we were computationally limited to 15,000 simulations containing unique parameter combinations using the quasi-random Sobol sequence number generation approach [82]. Each set of parameters feeds into one simulation. We removed simulations with fewer than 20 extant species as the patterns generated with too few species lack discriminatory power. Whilst still interesting, simulations containing very few species contained very little information on species-level diversity metrics. For example, if the simulation resulted in a few poorly distributed species, the diversity information regarding PD, MPD, and regional turnover resulting from under-developed diversity patterns adds quite a lot of noise to the analysis. This is the only filter applied, as we hoped to explore the parameter space as openly as possible. See Additional file 1: Fig. S3 for a comparison with Fig. 2, but retaining all simulations — the resulting patterns are largely the same. We compared the remaining simulations to real-world observed patterns of species richness aggregated to a 1-degree resolution [72]. The richness was therefore summed across all simulations which was then normalised, along with observed richness, between 0 and 1 to be comparable.

### Calculation of clade properties

Conceptually, we considered each simulation as representing a clade of fish with differing biological traits for which clade characteristics can be defined. These characteristics were calculated from the species object trait values and are summarised in Table 3. Our analyses comprise metrics at only the species-level, only the population-level, and at both levels.

**Table 3:** Summary of metrics

*Level*	*Metric*	*Description*
*Species*	Surviving species	The total number of extant species within a simulation.
	Species phylogenetic diversity	The total branch length in the phylogeny object, calculated using the phylomeasures R package [83, 84].
	Species mean pairwise distance	The mean pairwise distance between extant species in the phylogeny object, calculated using the phylomeasures R package [83, 85].
	Total species	The total number of extinct and extant species within a simulation.
	Species range	The mean number of occupied cells for all extant species.
	Species turnover	The number of extant species over the sum of extant and extinct species.
	Species richness	The mean simulation species richness per cell.
	Diversification rate	Calculated from the simulation phylogeny as the reciprocal of the evolutionary distinctiveness [86]. Evolutionary distinctiveness was calculated using the evol_distinct() function in the phyloregion R package [87] following the fair proportions framework [88].
	Weighted endemism	Weighted endemism for each cell was calculated as the number of species occupying that cell divided by the total ranges of those occupying species [89]. From this, the mean was taken.
*Population*	Total clusters	The total number of extant clusters of adjacent inhabited cells within all species in the simulation.
	Cluster phylogenetic diversity	Faith’s phylogenetic diversity [84] calculated from the population phylogeny.
	Cluster mean pairwise distance	The mean pairwise distance between populations in the population phylogeny.
*Both*	Continuity	The log-value of species diversity divided by the population diversity.
	Thermal traits	The mean, maximum, minimum, and range, evenness [90], and diversity [43].
	Competitive niche	The mean, maximum, minimum, and range, evenness [90], and diversity [43].

At the species-level we calculated the total number of species in a simulation, the total extant and extinct species across all time steps, species range size, temporal species turnover,

3 $$\begin{array}{c}Temporal\ species\ turnover = \frac{Extant\ species}{Extant\ species\ +\ Extinct\ species}\end{array}$$

and mean weighted endemism [89] per cell. Throughout the simulation, gen3sis calculates a species phylogeny based on pairwise species divergence times. From this species phylogeny we calculated global Faith’s phylogenetic diversity estimated as the total branch length within a phylogeny, and mean pairwise distance between species as the mean pairwise distance between objects within a phylogeny [85]. We calculated the diversification rate from the simulated phylogeny as the inverse of the evolutionary distinctiveness following the fair proportions framework [86, 88, 91]. As measures of functional trait diversity, we calculated the mean, maximum, minimum, range, evenness [43], and diversity [90] of the thermal and competitive niche traits using in-house functions in R [92].

At the population-level, we calculated the total number of geographic cell clusters per simulation (Additional file 1: Fig. S4) across all species as well as the phylogenetic diversity (PD) and mean pairwise distance (MPD). To calculate PD and MPD at the population-level, the divergence values between inhabited cells within each species were taken and aggregated into geographic clusters. The mean divergence value between each cluster is then calculated and decomposed into a cluster-to-cluster divergence matrix. A phylogeny object from this cluster divergence matrix was calculated using a hierarchical clustering approach implemented by hclust() in the R stats package [92]. From this cluster phylogeny, phylogenetic diversity is calculated using the pd() function in the phylomeasures R package [83]. The mean value from each simulation was then taken to make values comparable to the species-level phylogeny. Similarly, the mean pairwise distance was calculated as the mean pairwise distance between these geographic clusters of cells.

We focus on three different measures of diversity: richness, phylogenetic diversity (PD), and mean pairwise distance (MPD). Despite these metrics being conceptually related and occasionally correlated, they capture different aspects of biological diversity [57]. The relationship between the species and population levels of these diversity metrics, or the continuity across levels, was calculated. This was done by first normalising the constituent metrics across simulations (species richness/PD/MPD, cluster richness/PD/MPD) to between 0 and 1, making metrics relative measures across organisational levels. The species-level metrics were then divided by their corresponding cluster level metrics, e.g. species richness/cluster richness. These values were then log-transformed, giving positive values where species diversity was relatively higher than cluster diversity and negative values where it was lower. Formalised, this metric of continuity across levels was calculated as,

4 $$\begin{array}{c}Continuity=\mathrm{log}\left(\frac{species\ diversity}{population\ diversity}\right)\end{array}$$

This total diversity across simulations we defined as γ-diversity. To allow a β-diversity metric (geographic spatial comparisons) in our analyses, we divided the habitable cells in the model into bioregions, defined as realms by Spalding, Fox [93]; Central Indo-Pacific, Eastern Indo-Pacific, Tropical Atlantic, Tropical Eastern Pacific, and Western Indo-Pacific (Additional file 1: Fig. S5). Once subset into these bioregions, all diversity metrics described above were also calculated for each bioregion. β-diversity values are then the mean Euclidean distances between the continuity values amongst all pairs of bioregions in a simulation.

### Exploration of continuity patterns

We compared the relationship between the species and population levels of diversity in our simulations across the three facets of diversity: richness, phylogenetic diversity, and mean pairwise distance. For each facet comparison, a simple linear model was fit using the lm() function in the R stats package [92]. The models’ normal distribution assumption was satisfied using a log transformation for all diversity measures, except for species MPD. These continuity relationships were then investigated in light of biological parameter values: initial abundance, thermal optimum, dispersal distance, speciation threshold, mutation rate, and competitive niche width. For the continuity metrics of γ- and β-diversity, we fitted multiple linear regression models using the biological parameter values as predictors. These model variables were then reduced using a forward and backward stepwise model selection based on Akaike Information Criterion scores using the step() function in the R stats package [92]. Finally, we correlated the continuity metrics to the calculated clade properties: species range, thermal and niche trait evenness, weighted endemism, species turnover, and diversification rate. This was done with the hmisc package in R using Spearman’s Rank Correlation Coefficient to capture non-linear relationships between variables. *p*-values were Bonferroni corrected for multiple testing. This was visualised using a scaled PCA implemented in the R stats package [92].

### Supplementary Information

**Additional file 1: Figure S1.** Distribution of normalised diversity metrics at the species and population levels of organisation across retained simulations. **Figure S2.** Comparison of simulated and observed tropical fish species richness from Albouy, Archambault [72]. **Figure S3.** The results of main Figure 2, but without removing simulations with fewer than 20 surviving species. **Figure S4.** An example of population assignment in a simulation where each occupied cell has been clustered based on their dispersal distance and distance to one another. **Figure S5.** Assignment of simulation cells to the 5 tropical realms described by Spalding, Fox [93].

**Additional file 2:** **Table S1.** Summary of median diversity and clade properties. **Table S2.** Summary of multiple linear regression models predicting population-species level continuity using clade properties as predictor variables. **Table S3.** Correlation values between diversity continuity metrics and clade properties. *p*-values have been Bonferroni corrected. **Table S4.** Table of PCA contributions to each variable corresponding to visualisation in Figure 3b-d.

**Additional file 3:** Simulation output summary data. Table containing the metric values calculated for both all the cells in a simulation and for each realm described by Spalding, Fox [93]. This contains metrics from all simulations.

**Additional file 4:** **Figure 1.** data.

**Additional file 5:** **Figure 2.** data.

**Additional file 6: Figure 3.** data.

**Additional file 7:** **Figure S1.** data.

**Additional file 8: Figure S2. **data.

**Additional file 9: Figure S3.** data.

**Additional file 10: Figure S4.** data.

**Additional file 11: Figure S5.** data.

## Data Availability

All data generated or analysed during this study are included in this published article and its additional information files. All data and code are available online, here: https://doi.org/10.6084/m9.figshare.24548971. Data underlying figures and supplementary figures are available in the respective additional files referenced in the figure legends.

## Abbreviations

MPD: Mean pairwise distance
PD: Phylogenetic diversity
PCA: Principal component analysis
