# Supplementary material for: Diversity across organisational scale emerges through dispersal ability and speciation dynamics in tropical fish
Source: BMC Biol. 2023 Dec 5;21:282. doi: 10.1186/s12915-023-01771-3 (PMC10696697; doi:10.1186/s12915-023-01771-3)
Supplement: Supplementary file 1 — Additional file 1: Figure S1. Distribution of normalised diversity metrics at the species and population levels of organisation across retained simulations. Figure S2. Comparison of simulated and observed tropical fish species richness from Albouy, Archambault [72]. Figure S3. The results of main Figure 2, but without removing simulations with fewer than 20 surviving species. Figure S4. An example of population assignment in a simulation where each occupied cell has been clustered based on their dispersal distance and distance to one another. Figure S5. Assignment of simulation cells to the 5 tropical realms described by Spalding, Fox [93]. [file 12915_2023_1771_MOESM1_ESM.docx]

Diversity across organisational scale emerges through dispersal ability and speciation dynamics in tropical fish

# Additional file 1 Figures S1-S5


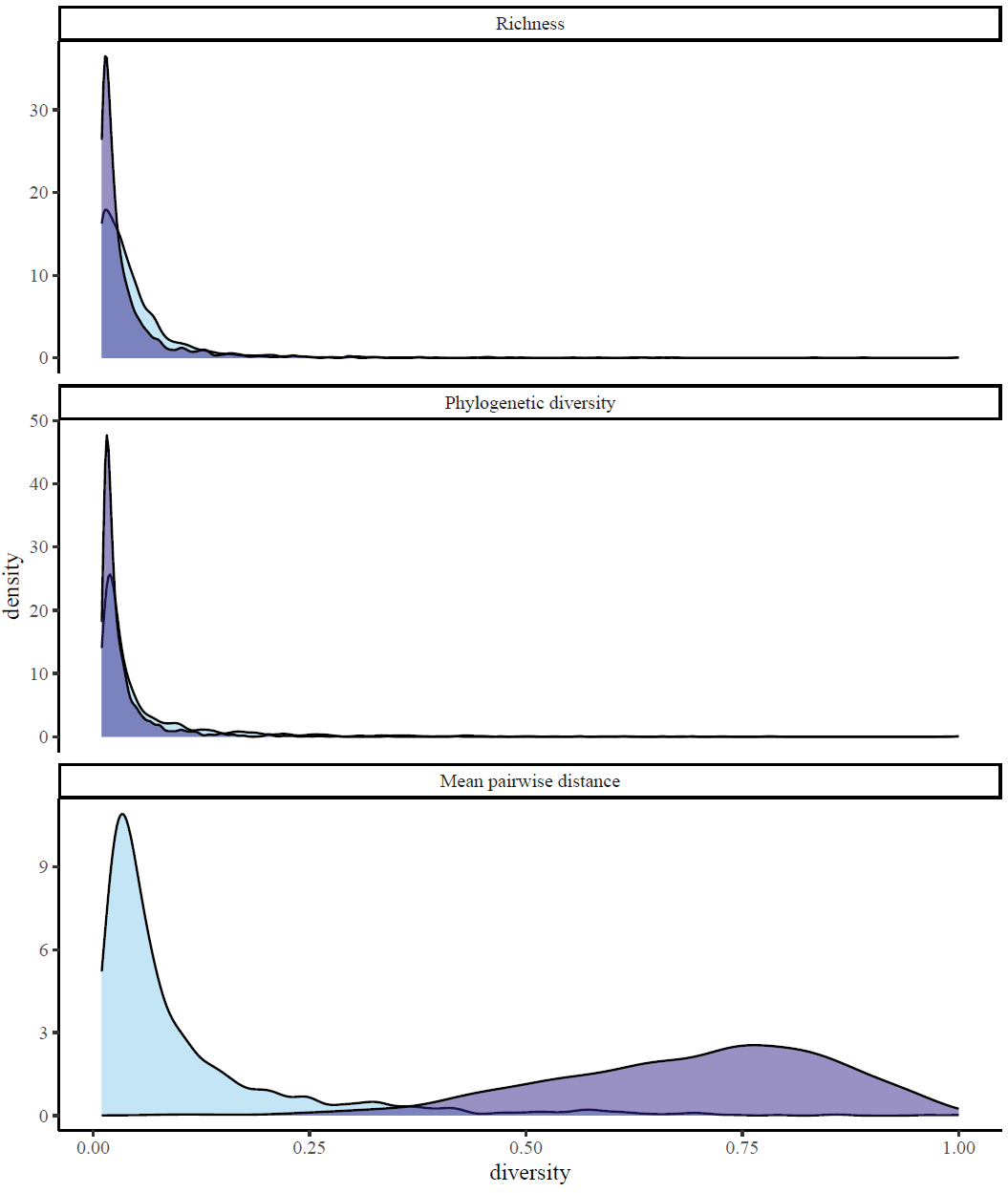


Figure S1: Distribution of normalised diversity metrics at the species and population levels of organisation across retained simulations. For the richness metrics (richness and phylogenetic diversity), the distributions are similar across levels. For the divergence metric (mean pairwise distance), the diversity distribution is heavily skewed to the right at the species level and skewed to the left at the population level. Figure data are available in Additional file 7.


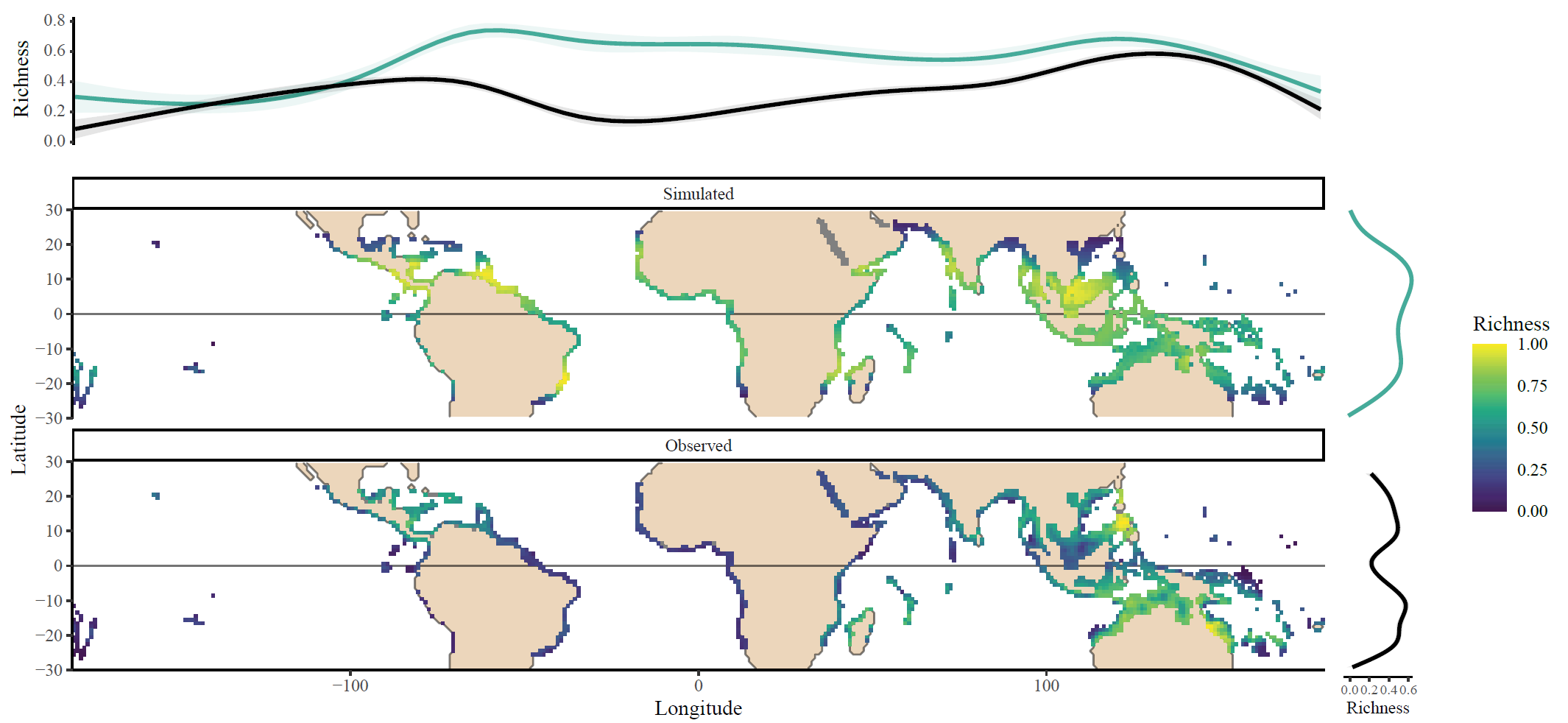


Figure S2: Comparison of simulated and observed tropical fish species richness. Simulated richness is the summation of all species present in all retained simulations. Observed richness is taken from Albouy et al. (72). Both datasets are normalised between 0 and 1 for comparability. Marginal plots are the mean richness values across latitude and longitude; green and black are simulated and observed, respectively. Grid cells are at 1° resolution. Figure data are available in Additional file 8.


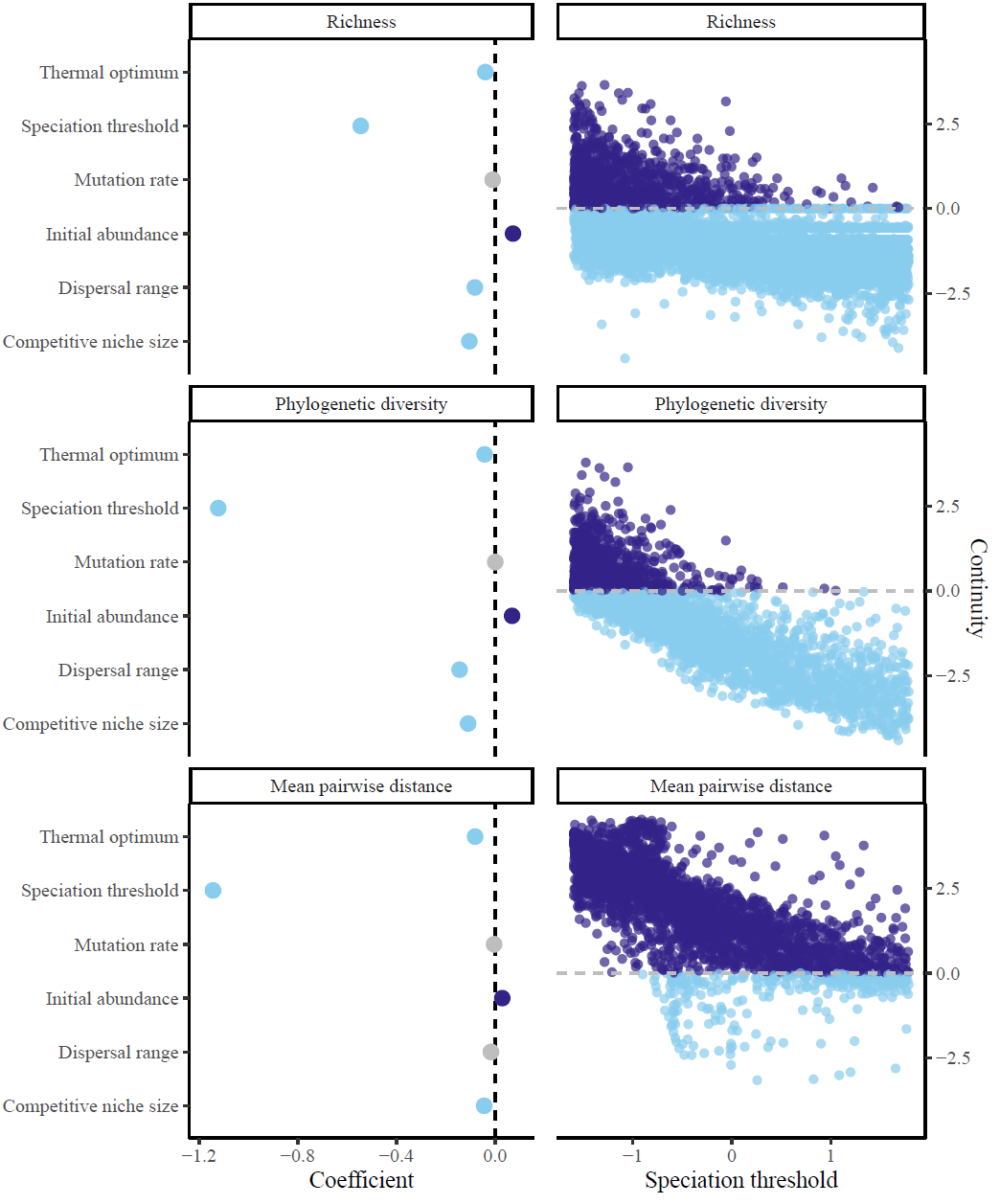


Figure S3: The results of main Figure 2, but without removing simulations with fewer than 20 surviving species. The results remain largely the same, but for the influence of initial abundance on continuity between the population and species levels in the mean pairwise distance diversity facet. Figure data are available in Additional file 9.


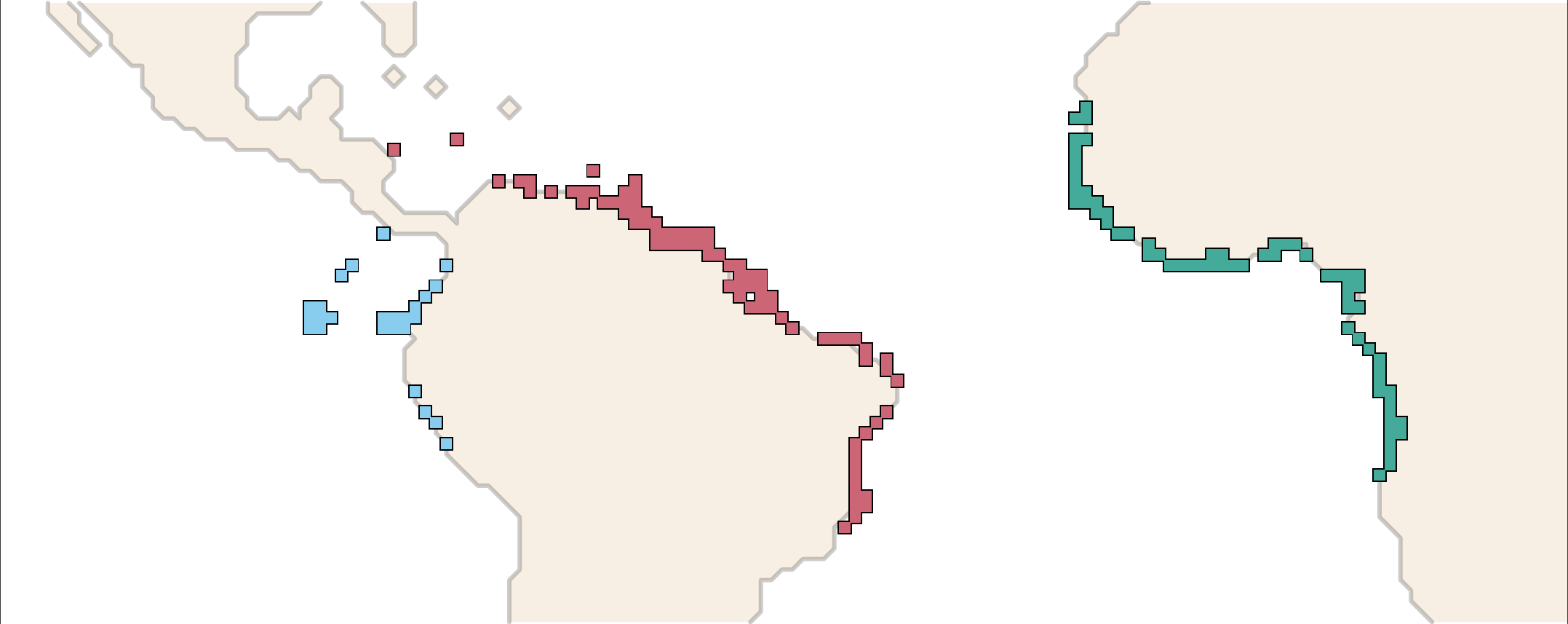


Figure S4: An example of population assignment in a simulation where each occupied cell has been clustered based on their dispersal distance and distance to one another. Each colour represents a geographic cluster which is then treated as the population object in the analyses. Each colour represents a geographic population. Figure data are available in Additional file 10.


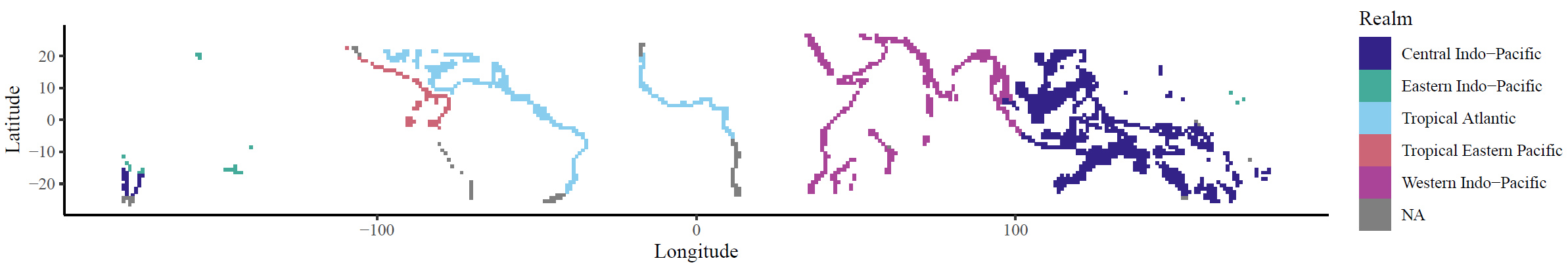


Figure S5: To calculate β-diversity, all habitable cells in each simulation were assigned to one of the 5 tropical realms described by Spalding et al. (90). Cells that were not assigned to a tropical realm were given no designation (NA values) and were discarded from the β-diversity analyses. Figure data are available in Additional file 11.
