# Supplementary material for: Diversity across organisational scale emerges through dispersal ability and speciation dynamics in tropical fish
Source: BMC Biol. 2023 Dec 5;21:282. doi: 10.1186/s12915-023-01771-3 (PMC10696697; doi:10.1186/s12915-023-01771-3)
Supplement: Supplementary file 2 — Additional file 2: Table S1. Summary of median diversity and clade properties. Table S2. Summary of multiple linear regression models predicting population-species level continuity using clade properties as predictor variables. Table S3. Correlation values between diversity continuity metrics and clade properties. p-values have been Bonferroni corrected. Table S4. Table of PCA contributions to each variable corresponding to visualisation in Figure 3b-d. [file 12915_2023_1771_MOESM2_ESM.docx]

Diversity across organisational scale emerges through dispersal ability and speciation dynamics in tropical fish

# Additional file 2

# Tables S1-S4

Table S1: Summary of median diversity and clade properties.

| Region | Species richness | Species PD | Species MPD | Population richness | Population PD | Population MPD |
| --- | --- | --- | --- | --- | --- | --- |
| Eastern Indo-Pacific | 9 | 7226.0000 | 1742.6571 | 1 | 1.8889 | 1.6719 |
| Tropical Eastern Pacific | 17 | 8015.0000 | 1725.7222 | 1 | 0.6853 | 0.5753 |
| Tropical Atlantic | 27 | 10897.0000 | 1731.3667 | 2 | 10.6790 | 7.7609 |
| Western Indo-Pacific | 27 | 10553.5000 | 1727.3862 | 2 | 15.8700 | 8.7944 |
| Central Indo-Pacific | 24 | 9777.0000 | 1721.7255 | 3 | 24.9884 | 11.9438 |
| **Global** | **55** | **16155.5000** | **1741.6552** | **3** | **35.4212** | **15.8218** |

| Region | Species range | Thermal evenness | Thermal diversity | Competitive evenness | Competitive diversity | Mean species richness | Weighted endemism | Species turnover | Diversification rate |
| --- | --- | --- | --- | --- | --- | --- | --- | --- | --- |
| Eastern Indo-Pacific | 4.8333 | 0.9405 | 0.0017 | 0.8643 | 0.0462 | 3.8636 | 0.0031 | 0.0168 | 0.0025 |
| Tropical Eastern Pacific | 18.6667 | 0.9937 | 0.0017 | 0.9287 | 0.0512 | 4.8281 | 0.0114 | 0.0542 | 0.0022 |
| Tropical Atlantic | 42.9857 | 0.9862 | 0.0016 | 0.9147 | 0.0527 | 4.5387 | 0.0128 | 0.0970 | 0.0028 |
| Western Indo-Pacific | 47.6875 | 1.0421 | 0.0015 | 0.9630 | 0.0497 | 3.9270 | 0.0053 | 0.0960 | 0.0029 |
| Central Indo-Pacific | 145.3333 | 1.0073 | 0.0013 | 0.9324 | 0.0485 | 4.4058 | 0.0032 | 0.0821 | 0.0027 |
| **Global** | **111.1812** | **1.0184** | **0.0015** | **0.9405** | **0.0508** | **4.3573** | **0.0064** | **0.1964** | **0.0038** |

Table S2: Summary of multiple linear regression models predicting population-species level continuity using clade properties as predictor variables.

|  | Richness Continuity | | | PD Continuity | | | MPD Continuity | | |
| --- | --- | --- | --- | --- | --- | --- | --- | --- | --- |
| Predictors | Estimates | Statistic | p | Estimates | Statistic | p | Estimates | Statistic | p |
| (Intercept) | 6.82 | 21.85 | **<0.001** | 7.74 | 34.70 | **<0.001** | 7.62 | 52.09 | **<0.001** |
| Dispersal range | -0.53 | -17.67 | **<0.001** | -0.41 | -19.16 | **<0.001** | -0.06 | -4.37 | **<0.001** |
| Speciation threshold | -0.79 | -23.99 | **<0.001** | -1.20 | -51.02 | **<0.001** | -1.09 | -70.52 | **<0.001** |
| Thermal optimum | -0.07 | -2.69 | **0.007** | -0.11 | -5.67 | **<0.001** | -0.10 | -8.23 | **<0.001** |
| Competitive niche size | -0.41 | -16.35 | **<0.001** | -0.26 | -14.71 | **<0.001** |  |  |  |
| Initial abundance | 0.30 | 11.84 | **<0.001** | 0.18 | 9.87 | **<0.001** | -0.04 | -3.11 | **0.002** |
| Observations | 1540 | | | 1540 | | | 1540 | | |
| R2 / R2 adjusted | 0.348 / 0.346 | | | 0.660 / 0.659 | | | 0.827 / 0.826 | | |

Table S3: Correlation values between diversity continuity metrics and clade properties. p-values have been Bonferroni corrected.

| **r values** | | | |
| --- | --- | --- | --- |
| Trait | *Richness* | *Phylogenetic diversity* | *Mean pairwise distance* |
| Species range | -0.14 | -0.15 | 0.00 |
| Thermal evenness | -0.47 | -0.42 | -0.17 |
| Thermal diversity | 0.22 | 0.19 | 0.04 |
| Competitive evenness | -0.49 | -0.48 | -0.26 |
| Competitive diversity | 0.42 | 0.47 | 0.27 |
| Weighted endemism | 0.12 | 0.09 | -0.09 |
| Species turnover | -0.33 | -0.78 | -0.86 |
| Diversification rate | 0.12 | -0.05 | 0.01 |

| **p values** | | | |
| --- | --- | --- | --- |
| Trait | *Richness* | *Phylogenetic diversity* | *Mean pairwise distance* |
| Species range | **0.00** | **0.00** | 47.41 |
| Thermal evenness | **0.00** | **0.00** | **0.00** |
| Thermal diversity | **0.00** | **0.00** | 4.83 |
| Competitive evenness | **0.00** | **0.00** | **0.00** |
| Competitive diversity | **0.00** | **0.00** | **0.00** |
| Weighted endemism | **0.00** | **0.03** | **0.03** |
| Species turnover | **0.00** | **0.00** | **0.00** |
| Diversification rate | **0.00** | 1.75 | 34.04 |

Table S4: Table of PCA contributions to each variable corresponding to visualisation in Figure 4b-d.

|  | ***Richness*** | | ***PD*** | | ***MPD*** | |
| --- | --- | --- | --- | --- | --- | --- |
| Variable | *Component 1* | *Component 2* | *Component 1* | *Component 2* | *Component 1* | *Component 2* |
| *Continuity* | 7.18 | 19.86 | 6.64 | 25.29 | 3.02 | 27.54 |
| *Species range* | 8.08 | 26.47 | 8.11 | 20.84 | 10.73 | 14.63 |
| *Thermal evenness* | 24.33 | 0.15 | 23.98 | 0.00 | 23.56 | 0.00 |
| *Thermal diversity* | 12.41 | 0.07 | 12.44 | 0.22 | 12.82 | 0.07 |
| *Competitive evenness* | 24.60 | 1.04 | 24.75 | 0.41 | 24.19 | 0.89 |
| *Competitive diversity* | 13.92 | 6.59 | 14.51 | 4.79 | 13.42 | 5.93 |
| *Weighted endemism* | 8.95 | 23.77 | 8.89 | 19.52 | 11.84 | 12.00 |
| *Species turnover* | 0.07 | 21.70 | 0.46 | 28.89 | 0.14 | 38.93 |
| *Diversification rate* | 0.43 | 0.35 | 0.21 | 0.04 | 0.27 | 0.01 |
